# Supplementary material for: The Inflammasome Activity of NLRP3 Is Independent of NEK7 in HEK293 Cells Co-Expressing ASC
Source: Int J Mol Sci. 2022 Sep 7;23(18):10269. doi: 10.3390/ijms231810269 (PMC9499477; doi:10.3390/ijms231810269)
Supplement: Supplementary file 1 [file ijms-23-10269-s001.zip › ijms-1850326-supplementary.pdf]

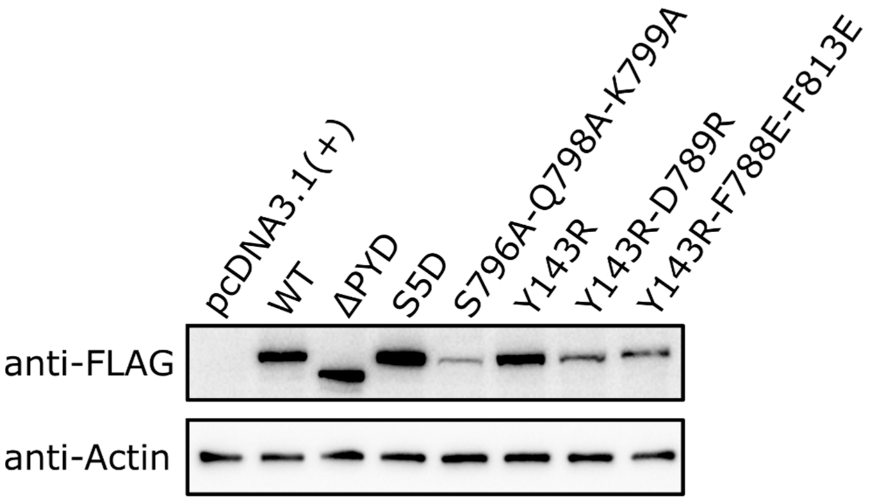

Supplementary Figure S1. Cell lysates of $7{\times}10^4$ HEK293 cells expressing wildtype or mutant NLRP3 were evaluated by anti-FLAG western blotting using a primary anti-FLAG antibody (Sigma #F1804) or a primary anti-pan-Actin antibody (Sigma #MAB1501) and a secondary HRP-conjugated anti-mouse antibody (ThermoFisher #32430).

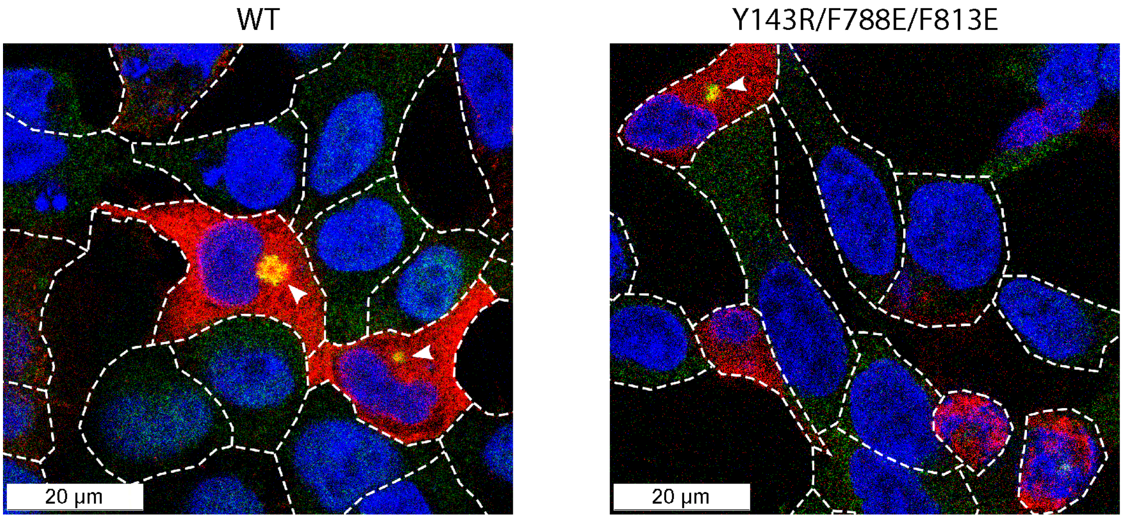

Supplementary Figure S2. Left: Two NLRP3-positive cells (red) expressing NLRP3-WT containing ASC specks (green, arrows) among several NLRP3-negative cells; the superposition of ASC and NLRP3 is apparent (yellow). Cell boundaries depicted by dashed lines. Right: Four NLRP3-positive cells expressing NLRP3-Y143R/F788E/F813E among several NLRP3-negative cells. Only one contains an ASC speck (arrow). Cell boundaries depicted as dashed lines.

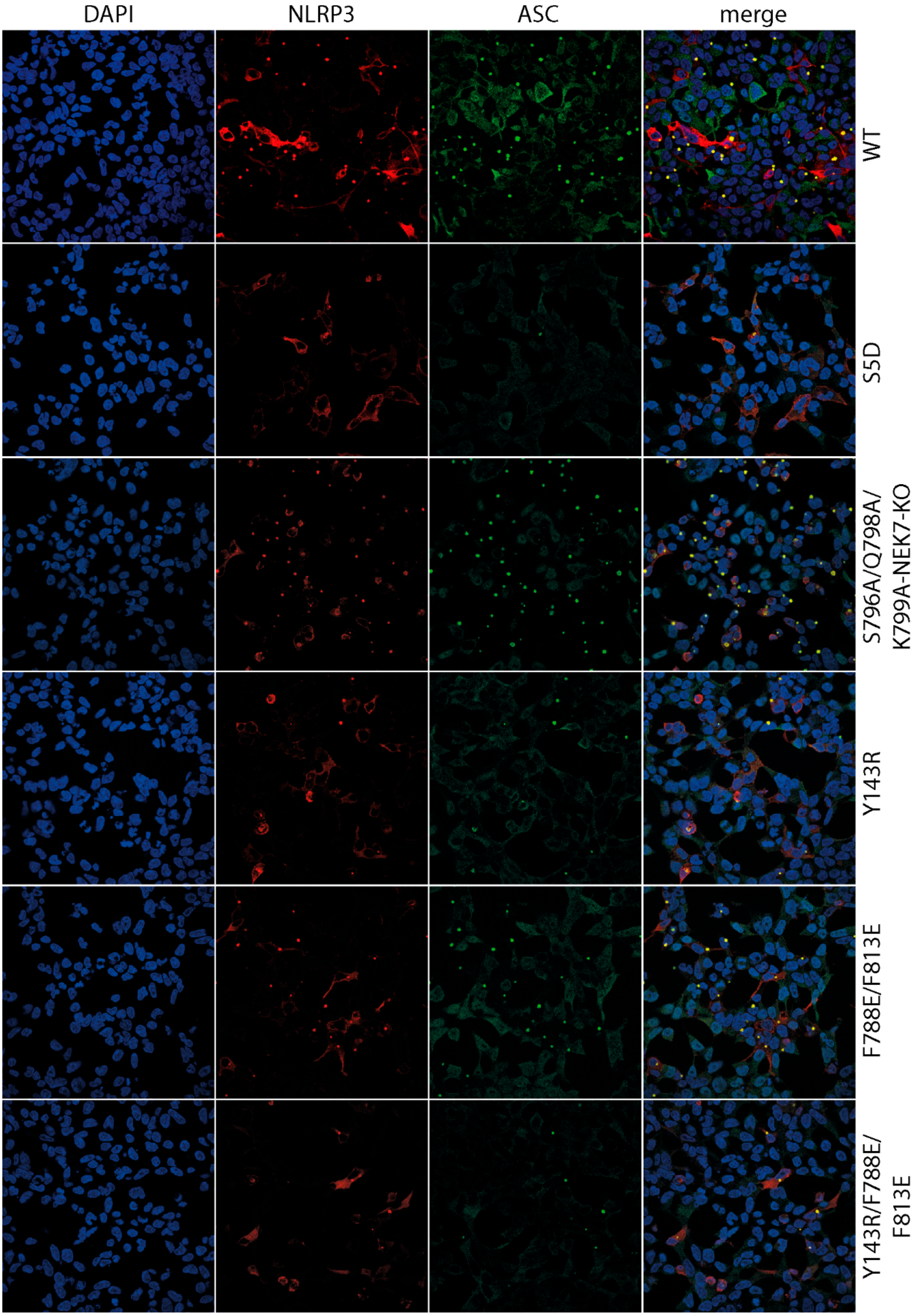

Supplementary Figure S3. Representative confocal fluorescence images of HEK293-ASC cells transfected with wild-type (WT) or mutant NLRP3-Flag. NLRP3-S796A-Q798A-K799A was transfected into NEK7-knockout cells. Primary antibodies against M2-Flag and Myc were used to detect NLRP3-Flag (red) and ASC-Myc (green). Nuclei were stained with DAPI (blue).

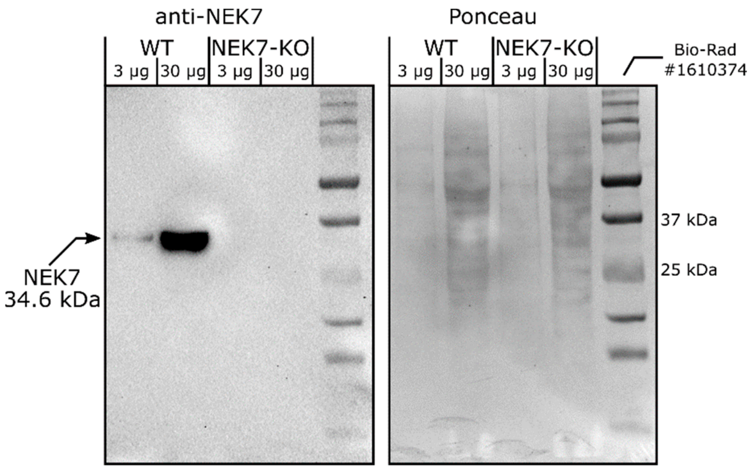

Supplementary Figure S4. NEK7-knockout in HEK293-ASC cells. Cell lysate with 3 or 30 µg total protein of wildtype or NEK7-KO cells was evaluated by anti-NEK7 western blotting using a primary anti-NEK7 antibody (abcam ab133514) and a secondary HRP-conjugated anti-Rabbit antibody (ThermoFisher #32460). Ponceau S coloration of total protein was performed to confirm successful loading and blotting.

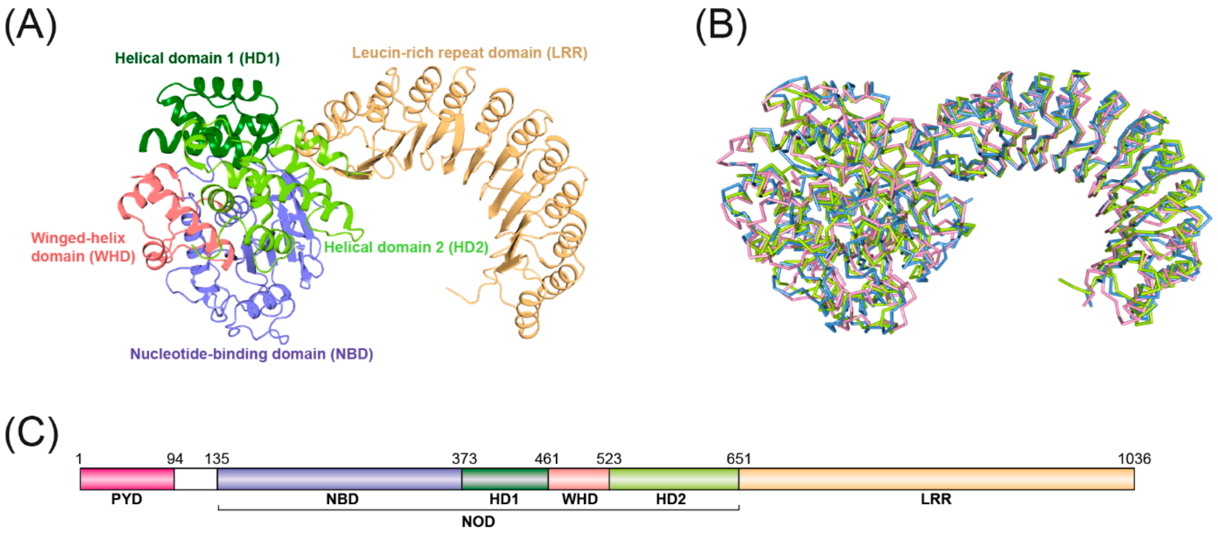

Supplementary Figure S5. Domain architecture of NLRP3. (A) Cartoon representation of a monomer of NLRP3ΔPYD taken from PDB ID 7PGU. The domains are color coded according to the labeling. (B) Cα traces of NLRP3ΔPYD monomers taken from the dodecamer (PDB ID 7FLH, shown in blue), the decamer (PDB ID 7PZC, shown in pink), and the hexamer (PDB ID 7PGU, shown in green) superposed onto each other. (C) Domain organization of NLRP3. Domains are color coded as in (A).
